# An Updated Meta-Analysis of Remote Blood Pressure Monitoring in Urban-Dwelling Patients with Hypertension

**DOI:** 10.3390/ijerph182010583

**Published:** 2021-10-09

**Authors:** Sang-Hyun Park, Jong-Ho Shin, Joowoong Park, Woo-Seok Choi

**Affiliations:** 1 Department of Internal Medicine, Daejeon Eulji Medical Center, Eulji University School of Medicine, Daejeon 35233, Korea; psh@eulji.ac.kr (S.-H.P.); redsea98@eulji.ac.kr (J.-H.S.); 2 Research Strategy Division, Korea Aerospace Research Institute (KARI), Daejeon 34133, Korea; park@kari.re.kr; 3 Moon Soul Graduate School of Future Strategy, Korea Advanced Institute of Science and Technology (KAIST), Daejeon 34141, Korea; 4 Keyu Internal Medicine Clinic, Daejeon 35250, Korea

**Keywords:** blood pressure, remote monitoring, hypertension, telemedicine, urban

## Abstract

Following the coronavirus disease-2019 pandemic, this study aimed to evaluate the overall effects of remote blood pressure monitoring (RBPM) for urban-dwelling patients with hypertension and high accessibility to healthcare and provide updated quantitative summary data. Of 2721 database-searched articles from RBPM’s inception to November 2020, 32 high-quality studies (48 comparisons) were selected as primary data for synthesis. A meta-analysis was undertaken using a random effects model. Primary outcomes were changes in office systolic blood pressure (SBP) and diastolic blood pressure (DBP) following RBPM. The secondary outcome was the BP control rate. Compared with a usual care group, there was a decrease in SBP and DBP in the RBPM group (standardized mean difference 0.507 (95% confidence interval [CI] 0.339–0.675, *p <* 0.001; weighted mean difference [WMD] 4.464 mmHg, *p <* 0.001) and 0.315 (CI 0.209–0.422, *p <* 0.001; WMD 2.075 mmHg, *p <* 0.001), respectively). The RBPM group had a higher BP control rate based on a relative ratio (RR) of 1.226 (1.107–1.358, *p <* 0.001). RBPM effects increased with increases in city size and frequent monitoring, with decreases in intervention duration, and in cities without medically underserved areas. RBPM is effective in reducing BP and in achieving target BP levels for urban-dwelling patients with hypertension.

## 1. Introduction

Hypertension is widely recognized as the most important risk factor for cardiovascular disease (CVD), which is a major cause of total mortality [1]. A 2 mmHg fall in systolic blood pressure (SBP) has been reported to reduce the incidence of ischemic CVD and stroke by 7% [2]. However, even in advanced countries, target blood pressure (BP) is achieved in <50% of patients with hypertension [3,4]. The 2017 American College of Cardiology/American Heart Association (ACC/AHA) and 2018 European Society of Cardiology/European Society of Hypertension (ESC/ESH) treatment recommendations state that BP must be controlled to stricter levels [5,6]. 

Remote BP monitoring (RBPM) has been recommended for hypertension diagnosis and treatment [5,6], as it has been reported to predict CVD morbidity and mortality with higher accuracy than office BP monitoring [7]. As a method of telemedicine, RBPM is known to be an effective tool to enhance drug adherence and BP control in patients with hypertension [8,9,10,11,12]. RBPM has been suggested as a potential solution to overcome the geographical limitations of healthcare services [13], with significant effects shown in randomized controlled trials (RCTs) and meta-analysis studies [10,14,15,16]. The 2017 ACC/AHA guidelines also recommended RBPM for hypertension diagnosis and control, and for enhancing patients’ drug adherence [6].

According to the United Nations, approximately 68% of the human population is predicted to dwell in urban settings by 2050 [17]. Urbanization is a rapidly growing 21st century trend, with significant effects on human health. However, despite increased interest in new health technologies, several studies have reported that remote monitoring has limited application in urban settings where high-quality face-to-face care is possible and healthcare accessibility is high [18,19]. Moreover, there is no comprehensive evidence concerning the effect of RBPM in improving clinical outcomes of urban-dwelling patients with hypertension or whether RBPM can become a standard treatment for hypertension management.

In a previous meta-analysis of RCTs using the Jovell/Navarro-Rubio classification system to determine the strength of evidence, RBPM showed statistically significant reductions in SBP (3.48 mmHg) and diastolic BP (DBP, 1.64 mmHg) compared with usual care (UC) after an average of 7.6 months for patients dwelling in an urban setting. In terms of CVD prevention, however, RBPM induced <0.5% of CVD prevention in low-risk patients with hypertension. Therefore, some studies have concluded that RBPM is of little practical significance to policy-makers [20,21]. The coronavirus disease-2019 (COVID-19) pandemic resulted in a steeply increased demand for telemedicine, even in urban settings, for those otherwise having adequate availability and accessibility to healthcare services. More generally, characteristically dense populations in cities have resulted in the rapid spread of infectious diseases, leading to the expansion of infrastructure for non-face-to-face care in line with a rapid increase in the use of the internet and mobile devices.

Considering the global rate of BP control, according to 2017 ACC/AHA guidelines for hypertension diagnosis and control, which is the latest strict guideline for hypertension diagnosis and control, the proportion of patients achieving the target BP is predicted to decrease further. The use of remote medical care services suddenly increased during the COVID-19 pandemic [14,22,23], and its use needs to be verified based on the integration of previous findings, given that hypertension is a chronic disease requiring long-term management for CVD prevention and for efficient healthcare policies to be implemented in urban settings. Therefore, relevant studies need to be extended through an updated compilation of BP data. The objective of our study is to evaluate whether RBPM could be utilized as an alternative to standard treatment for urban-dwelling patients with hypertension during the COVID-19 pandemic. Thus, this study aimed to determine the relative effects of RBPM compared with UC based on outcomes including SBP, DBP, and BP control rates. Intervention duration, city size, setting, frequency of remote transmission of BP data, and the presence of medically underserved areas (MUAs) in the city were analyzed as secondary factors to evaluate the effects of RBPM. We hypothesized that the effects of RBPM were equivalent to those of UC. To test this hypothesis, relevant, up-to-date RCTs were systematically reviewed and transparent and reliable quantitative data synthesis was performed.

## 2. Materials and Methods

### 2.1. Searching for Eligible Studies

This study followed the Preferred Reporting Items for Systematic Reviews and Meta-Analyses (PRISMA) guidelines of the Cochrane Collaboration and a checklist was provided [Supplementary Materials] [24]. To identify eligible studies, two investigators (SHP and JHS) independently searched the following electronic databases: PubMed, EBSCOhost, Embase, and the Cochrane Library, from RBPM’s inception to November 30, 2020. Free terms were used, including and related to *urban*, *hypertension*, and *remote monitoring*, along with medical subject heading (MeSH) terms. Truncation and phrasing methods were applied to derive a structured search formula [20] (Appendix A). The formula was first applied to the Cochrane Library and then converted to suit each database for the subsequent search. Articles written in English were retrieved. To include as many relevant articles as possible, all systematic reviews and meta-analyses related to the search themes were collected from each database and Google Scholar, and their reference lists were reviewed. To identify gray literature, relevant websites were used, and all studies including those in which the city area was not clearly defined were identified through a manual search.

### 2.2. Inclusion and Exclusion Criteria

All included studies were blinded RCT studies with random and uniform allocation of patients with hypertension into an RBPM group and a traditional face-to-face UC group. Articles reporting pre- and post-intervention data were targeted, with participants satisfying the following criteria: (1) patients with hypertension under management through regular visits to an urban medical institution; (2) patients able to measure their own BP at home; (3) patients able to transmit their BP data to the physician via post, phone, Bluetooth device, mobile phone, web, or computer (wired or wireless); (4) adults aged ≥18 years; (5) BP measurement through ambulatory monitoring; and (6) various transmission methods from real-time or a stored and forward method to an automatic or manual method. Exclusion criteria comprised the following: (1) sudden BP changes due to an acute CVD or cerebrovascular accident (CVA); (2) patients undergoing hemodialysis due to acute or chronic renal disease; (3) female patients before and after pregnancy; (4) cases not reported for urban areas or cases for urban and rural areas reported together; (5) cases from unclear target areas; (6) cases where monitoring was aided by medical staff at a nursing management unit or care facility; and (7) cluster trials or cross-over studies.

### 2.3. Study Selection

The citations retrieved from each database were exported to EndNote X8.2, and two investigators (SHP and JHS) independently eliminated those not satisfying the criteria to confirm the reliability of identification. First, the title and abstract were screened, and for studies satisfying the criteria, full texts were obtained and scrutinized. Primary studies were selected independently, and their reference lists were reviewed. Final articles for data synthesis were determined after discussion with the senior author (WSC).

### 2.4. Data Extraction and Coding

For the selected studies, data extraction was performed independently by two investigators (JHS and WSC), and relevant values were coded in an electronic sheet. The extracted data included demographic and pre- and post-intervention SBP and DBP data. BP data were mostly obtained using an automated device and, in the case of ambulatory BP monitoring (ABPM), the mean of each group was calculated and coded. If an article did not report BP values or standard deviations (SDs), preventing calculations with a 95% confidence interval, the values were first checked on the trial registries website and, in cases where the required information could not be obtained, an attempt was made to contact the author of the article [25,26]. Articles that satisfied the inclusion criteria but did not report the main BP data were excluded from the final data synthesis. For some studies with missing SDs, data imputation was performed using a simple method [27,28]. The mean of all other studies, excluding those with missing data, was obtained. Regarding the rate of BP control, the number of patients satisfying the level of normal BP, determined during the final follow-up period of comparison in each study, was calculated and compared between the two groups. If a single primary study included several different follow-up periods for comparison [26,27,28,29,30,31,32,33,34,35]; applied a different, additional intervention [25,34]; or had multiple varying sample sizes and thus reported varying results, each result was included in the analysis as an independent study. Disagreements between investigators were resolved through consultation with the senior author (WSC).

### 2.5. Quality Assessment and Publication Bias

The quality assessment of the primary studies included evaluating the risk of bias (RoB) and was performed independently by two investigators (SHP and JP). Using the Review Manager program (RevMan, version 5.3.5, Copenhagen, Denmark) software from the Cochrane Collaboration, the evaluation was performed according to the Cochrane Handbook for Systematic Reviews of Interventions guidelines [24,36]. Disagreements were resolved through discussion among investigators. To identify publication bias, Egger’s regression, classic fail-safe N, Duval and Tweedie’s trim-and-fill method, and funnel plots were used.

### 2.6. Statistical Analysis

**Figure A1:**
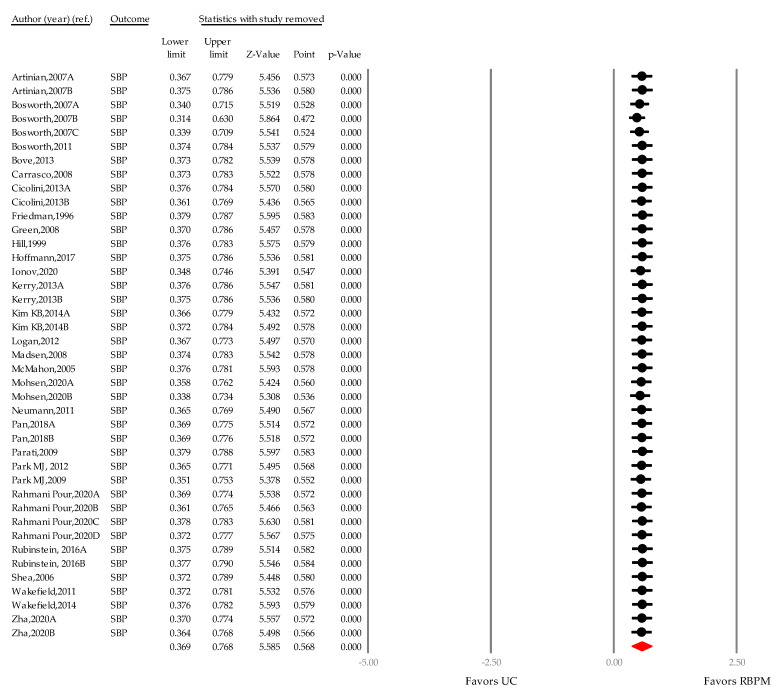
Note: point estimate of individual study (●), summary effect size (◆); SBP, systolic blood pressure; UC, usual care; RBPM, remote blood pressure monitoring.

**Figure A2:**
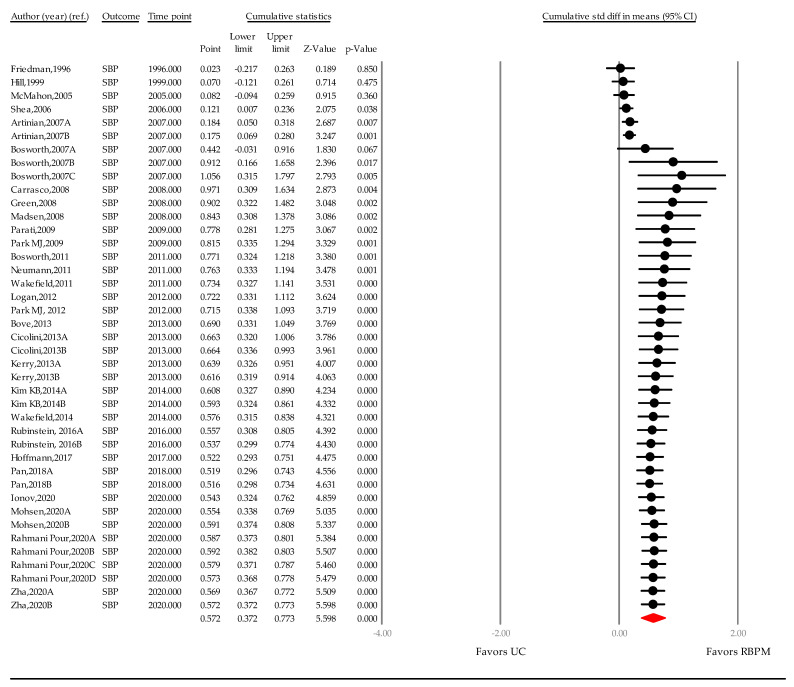
Note: point estimate of individual study excluding each individual study (●), summary effect size (◆); SBP, systolic blood pressure; SMD, standardized mean difference; UC, usual care; RBPM, remote blood pressure monitoring.

To ensure the reliability of the analysis, coded data were analyzed by two investigators (SHP & JHS) using Comprehensive Meta-Analysis version 2 (CMA, Biostat, Englewood, NJ, USA) software. For primary outcomes, continuous variables comprised the weighted mean difference (WMD) and the standardized mean difference (SMD) obtained from the mean SBP and DBP values measured at baseline and during follow-up in the office. Despite divided opinions regarding the use of continuous variables, SMD has shown a trend of higher statistically significant generalizability and percentage agreement than the WMD in a random effects model (REM) and a fixed effects model (FEM) [37,38]. Therefore, SMD was used in this study to report the results of the data synthesis for continuous variables. Considering the generalizability of each result, the WMD was additionally estimated for comparing the subgroup results [38]. Based on Cohen’s general rule of thumb, the effect size was set as follows: SMD 0.2 (small effect); SMD 0.5 (medium effect), and SMD 0.8 (large effect) [39]. Accordingly, when the SMD was ≥0.5, we considered the effect size to be significant in this study. The rate of BP control was a dichotomous variable, for which BP normalization data were extracted from each study, and effect size based on relative risk (RR) was used. A 95% confidence interval (CI) was used for all data. To analyze the inter-rater difference, a χ^2^ test was used and the level of significance was set to *p <* 0.10. The model of analysis was applied after assessing the enrolled population of each study and the heterogeneity among research centers. Between-study heterogeneity was presented using Tau-squared (τ^2^) and I-squared (I^2^) indices, and the adequacy of results was determined based on Cohen’s general rule of thumb [40]. Therefore, in this study, 30 ≤ I^2^ ≤ 60 indicated moderate heterogeneity and 50 ≤ I^2^ ≤ 90 indicated substantial heterogeneity [39]. To assess the quality of each trial and the consequent impact on the overall effect size, sensitivity was tested using the “one study removed” method (Appendix B, Figure A1). A cumulative analysis was run for a total of 48 comparisons, and the range of summary effect sizes at each step according to temporal progression was determined. *p*-values and the presence of outliers affecting the overall effect size were also determined (Appendix C, Figure A2). An additional sensitivity test was performed to determine differences between the data before and after imputing the missing values.

## 3. Results

### 3.1. Study Characteristics

**Figure 1:**
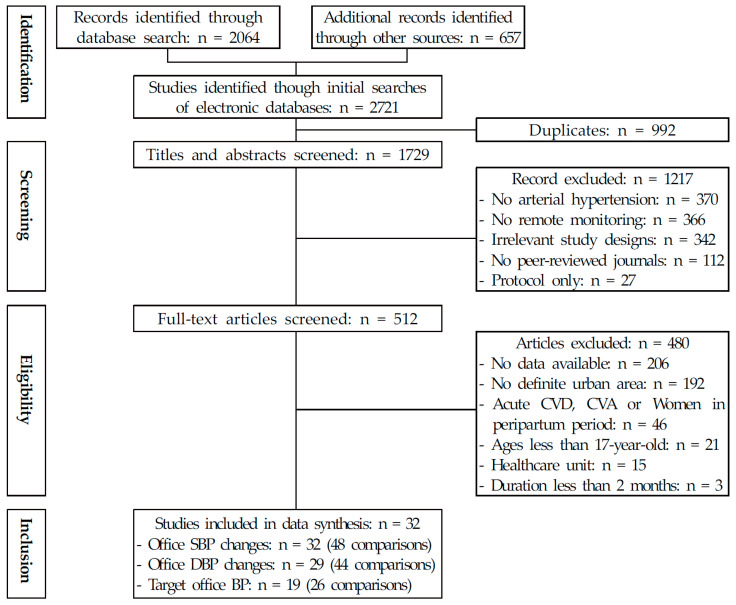
PRISMA flow of study. Abbreviations: BP, blood pressure; CVD, cardiovascular disease; CVA, cerebro-vascular accident; DBP, diastolic blood pressure; SBP, systolic blood pressure.

Through an initial search of available databases, reference to trial registries, and a manual search of reference lists, a total of 2721 citations were retrieved (Figure 1). Of these, 992 duplicates were removed, leaving 1729 citations to be identified. Next, titles and abstracts for each identified citation were screened, and 1217 irrelevant citations were excluded. For the remaining 512 articles, the full text was obtained and scrutinized, and studies without available data (*n* = 206), studies not performed in an urban area, studies either reporting combined results of urban and rural areas or not reporting the area (*n* = 192), studies conducted on patients with CVD or CVA that may induce a sudden change in BP, studies conducted on patients undergoing hemodialysis or including patients with chronic renal disease, and studies involving female patients before or after pregnancy (*n* = 46) or patients aged <18 years (*n* = 21) were excluded. In total, 32 independent studies (48 comparisons) satisfying the inclusion criteria were used in the final data synthesis (Table 1).

For the primary studies included in the meta-analysis in this study, the duration of RBPM was 2–18 months (mean, 7.37 months), and the number of participants in the UC and RBPM groups was 5666 and 5729, respectively. The mean age of participants in the UC and RBPM groups was 52.63 and 52.17 years, respectively. No significant intergroup differences were found in terms of sex and baseline BP. No differences in ethnicity were observed. Fourteen studies were conducted in primary medical institutions, 12 in community healthcare centers, and 22 in hospitals or higher-level institutions. The completion dates were in or prior to the year 2000 for two studies [41,42], between 2001 and 2010 for 14 studies [25,29,43,44,45,46,47,48,49,50], and between 2011 and 2020 for 32 studies. Seven studies had used mean values for ABPM [47,48,50,51,52,53,54].

### 3.2. Risk Assessment

To check for bias in RCT studies, the Cochrane Group’s RoB tool of the Cochrane group was used for domain analysis based on a checklist. Across seven domains, a low risk of selection bias related to sequence generation or allocation concealment was shown. Similarly, the risk of detection bias related to blinding of personnel and patients was appropriately reported. Concerning attrition bias (incomplete outcome data), an unclear or sufficiently high risk was shown that raised concern in a number of studies; however, as most studies showed a low risk (≥4) across the seven domains, the overall RoB was deemed to be low [62].

**Figure 2:**
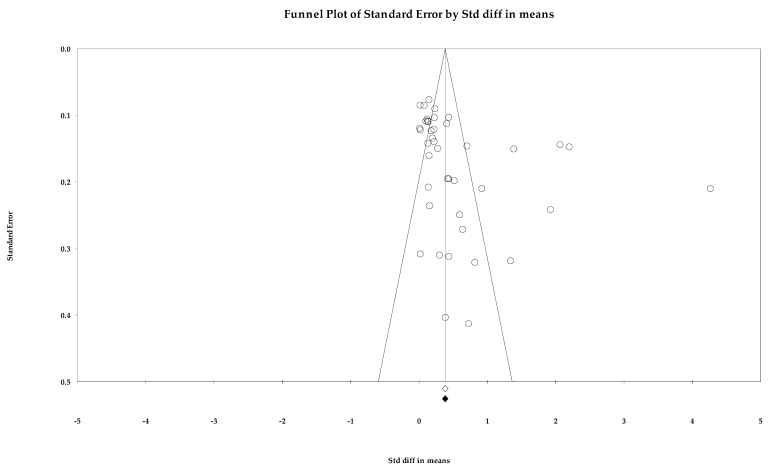
A funnel plot of the standardized mean difference in systolic blood pressure. Note: summary effect size (◇), summary effect size of imputed studies (◆), individual study (○).

**Figure 3:**
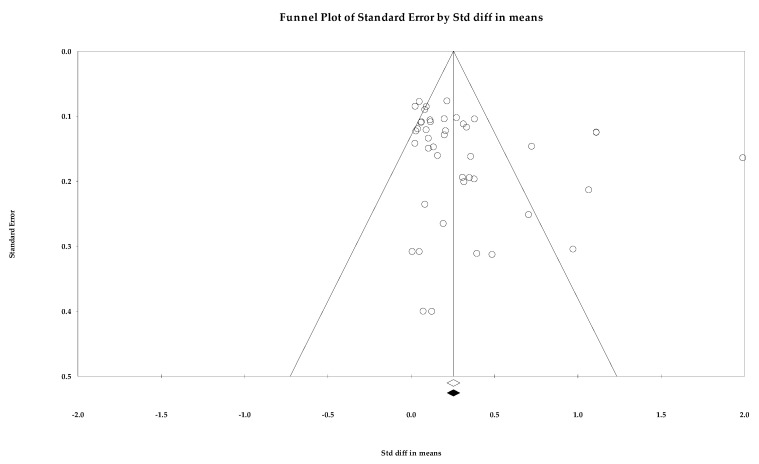
A funnel plot of standardized mean difference in diastolic blood pressure. Note: summary effect size (◇), summary effect size of imputed studies (◆), individual study (○).

**Figure 4:**
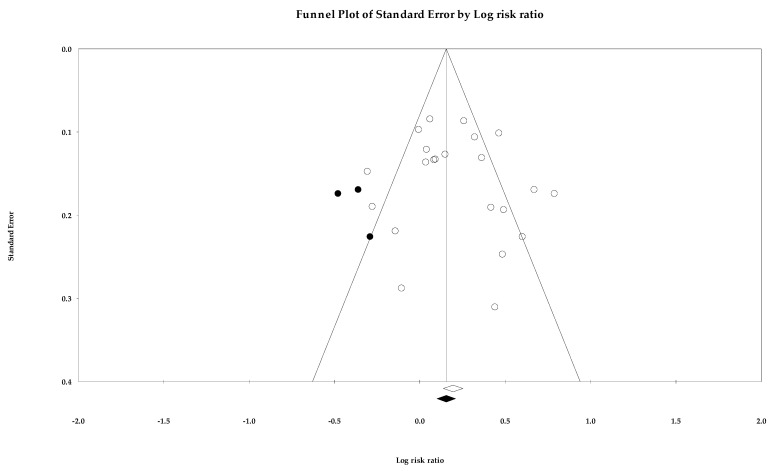
A funnel plot of relative risk for the target blood pressure rate. Note: summary effect size (◇), imputed study (●), summary effect size of imputed studies (◆), individual study (○).

Egger’s regression intercept was 4.516 (1.363–7.669; *p =* 0.005) in two-tailed 95% CIs [37]. The number of studies needed to attain *p >* 0.05 for a classic fail-safe N was 5085. The point estimate of SBP in Duval and Tweedie’s trim-and-fill analysis (SMD, 0.507 mmHg (0.339–0.645, *p <* 0.001); WMD, 4.464 mmHg (*p <* 0.001)) coincided with the summary effect size, while no imputed study was found in the funnel plot (Figure 2) [63]. The SMD of DBP was 0.253 (0.215–0.292), and no study was trimmed (Figure 3). In the analysis of the rate of target BP achievement, RR was 1.237 (1.107–1.381), three studies were imputed, and the adjusted value was 1.161 (1.032–1.306, Figure 4). Although RoB assessment detected a certain level of publication bias, the overall data were statistically significant and the analysis results were not rejected.

A sensitivity test was performed for studies that had been included to prevent small-study effects, excluding those with a sample size of ≤50 for the RBPM group [64]. The test results showed an SMD of 0.501 mmHg (0.313–0.689, *p <* 0.001) and a WMD of 4.238 mmHg (*p <* 0.001), indicating that the difference from the overall summary effect size was not clinically significant and that the potential small-study effect was not significant in this study.

### 3.3. Primary Outcomes

#### 3.3.1. Systolic Blood Pressure 

**Figure 5:**
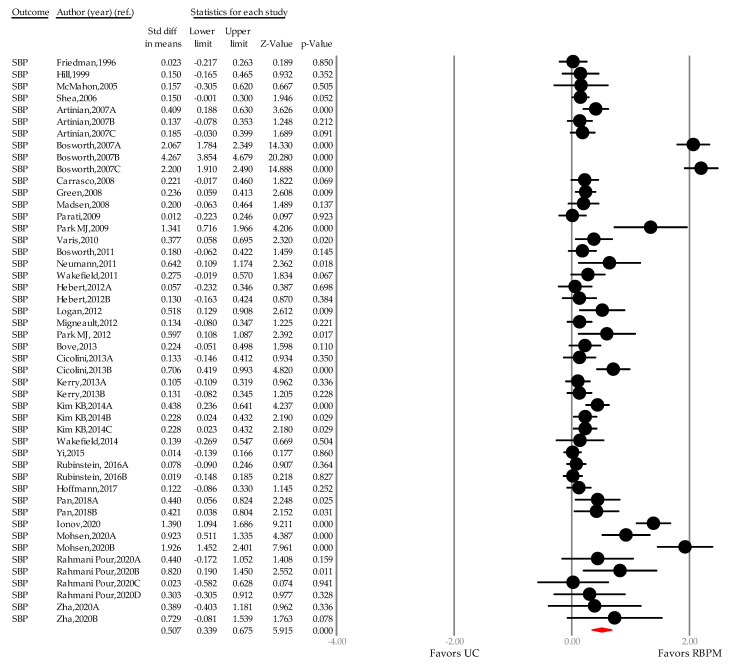
A forest plot of standardized mean difference in systolic blood pressure. Note: point estimate of individual study (●), summary effect size (◆); SBP, systolic blood pressure; UC, usual care; RBPM, remote blood pressure monitoring.

Across 32 independent studies (48 comparisons), 11,395 patients (UC group, *n* = 5666; RBPM group, *n* = 5,729) were analyzed for SBP [25,26,27,28,29,30,31,32,33,34,35,41,42,43,44,45,46,47,48,49,50,51,52,53,54,55,56,57,58,59,60,61]. The summary SMD was 0.507 (0.339–0.675, *p <* 0.001), showing an above moderate effect size, and the WMD after conversion was 4.464 mmHg (3.371–5.556, *p <* 0.001; Figure 5). The between-group heterogeneity was significant (I^2^ = 70.908%, *p <* 0.001). To determine the effect of individual studies on the total summary effect size, a sensitivity test was performed using the “one study removed” method, whereby each study was sequentially omitted (Appendix B). Here, the point estimate of the summary effect size showed no significant difference and no outliers were detected.

When the average effect of RBPM was chronologically divided into three timeframes and compared with the UC group (Phase I, inception of RBPM to 2000; phase II, 2001–2010; phase III, 2011–2020), the WMD was 1.515 mmHg (*n* = 2, −4.031–7.061, *p =* 0.592; I^2^ = 0.000%, *p =* 0.478) in phase I [41,42], 4.333 mmHg (*n* = 14, 2.338–6.328, *p <* 0.001; I^2^ = 38.554, *p <* 0.001) in phase II [25,29,43,44,45,46,47,48,49,50], and 4.719 mmHg (*n* = 32, 3.343–6.094, *p <* 0.001; I^2^ = 77.361%, *p <* 0.001) in phase III [26,27,28,30,31,32,33,34,35,51,52,53,54,55,56,57,58,59,60,61]. 

#### 3.3.2. Diastolic Blood Pressure

**Figure 6:**
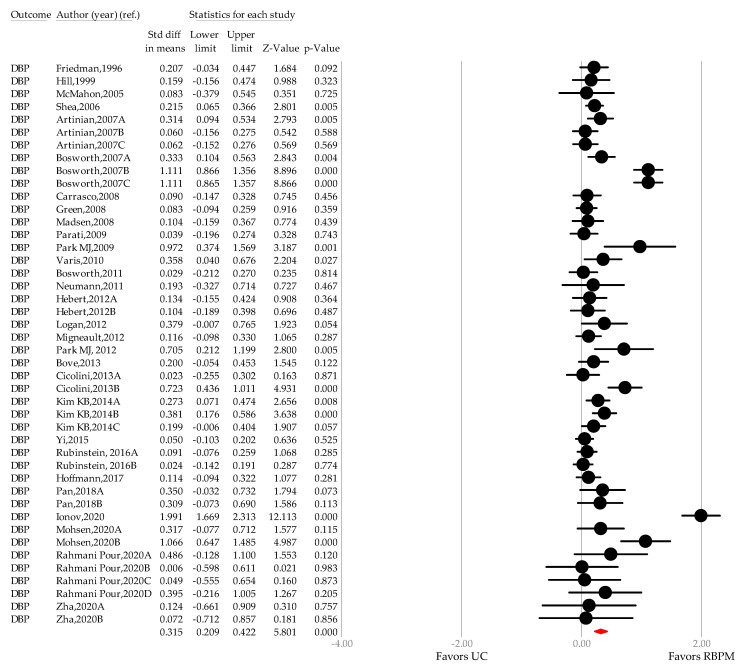
A forest plot of standardized mean difference in diastolic blood pressure. Note: point estimate of individual study (●), summary effect size (◆); DBP, diastolic blood pressure; UC, usual care; RBPM, remote blood pressure monitoring.

To determine the effect of RBPM on DBP, data concerning 10,482 patients (UC group, *n* = 5192; RBPM group, *n* = 5290) were analyzed across 29 studies (44 comparisons) [25,27,28,29,30,31,32,33,34,35,41,42,43,44,45,46,47,48,49,50,51,52,53,54,56,57,58,59,61]. Compared with the UC group, the RBPM group showed greater BP reduction (SMD, 0.315 mmHg (0.209–0.402), *p <* 0.001; WMD, 2.075 mmHg (1.399–2.750) *p* < 0.001) after conversion (Figure 6). The between-study heterogeneity was substantial (I^2^, 68.021%; *p <* 0.001). No outliers were detected in the sensitivity test performed through sequentially omitting each study.

The WMD according to time interval was 2.059 mmHg in phase I (*n* = 2, −1.143–5.262, *p =* 0.208; I^2^ = 0.000%, *p =* 0.45)[41,42], 1.587 mmHg in phase II (*n* = 14, 0.421–2.753, *p <* 0.001; I^2^ = 17.407%, *p <* 0.001) [25,29,43,44,45,46,47,48,49,50], and 2.348 mmHg in phase III (*n* = 28; 1.480–3.216, *p <* 0.001; I^2^ = 76.230%, *p <* 0.001) [26,27,28,30,31,32,33,34,35,51,52,53,54,55,56,57,58,59,60,61]. 

#### 3.3.3. Target Blood Pressure Rate

**Figure 7:**
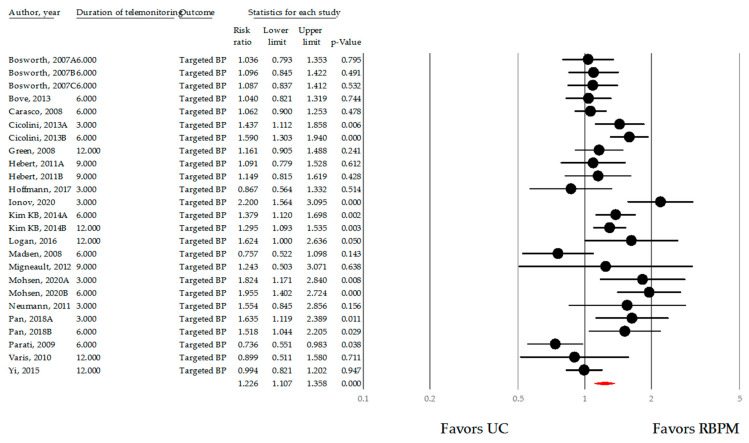
Risk ratio of target blood pressure using remote blood pressure monitoring. Note: point estimate of individual study (●), summary effect size (◆); BP, blood pressure; UC, usual care; RBPM, remote blood pressure monitoring.

To determine the effect of RBPM, the rate of BP control was estimated based on BP normalization criteria defined in each primary study. Across 16 studies (25 comparisons), the data of 2655 patients in the UC group and 2816 patients in the RBPM group were comprehensively analyzed [13,25,30,31,32,33,45,46,47,50,51,52,53,59,61]. Compared with the UC group, the RBPM group showed a significant effect, with an approximately 23.7% higher improvement in BP control based on RR (RR= 1.226 (1.107–1.358), *p <* 0.001; Figure 7). The between-study heterogeneity was substantial (I^2^ = 70.656%; *p <* 0.001). No significant difference in summary effect size was found in the sensitivity test.

### 3.4. Subgroup Analysis

#### 3.4.1. City Size 

Generally accepted international criteria define city size according to population size in a given area. In this study, a metropolitan city was defined as a city with a population of at least one million. Thus, the RCT studies included in this study were categorized based on city size as either a small-to-medium-sized city study or a large city study, and the two categories were analyzed separately. Population size was estimated from the data of the latest international population survey performed in the nearest period of time to this study. Of the 48 studies, 22 were conducted in small-to-medium cities [25,28,29,32,41,42,43,46,47,50,51,54,55,56,57] and 26 were conducted in large cities [26,27,30,31,33,34,35,44,45,48,49,52,53,58,59,60,61]. For the former, the SBP showed a WMD of 3.860 mmHg (2.271–5.450, *p <* 0.001) without between-study heterogeneity (I^2^ = 0.000, *p =* 0.478; Tau^2^ = 0.000). For the latter, the SBP showed a WMD of 5.056 mmHg (3.503–6.609, *p <* 0.001) with a significant level of between-study heterogeneity (I^2^ = 82.177%, *p <* 0.001, Tau^2^ = 17.368); the magnitude of the effect size was above moderate. 

#### 3.4.2. Medically Underserved Areas

The presence of MUAs for each group was reflected in the analysis only if the study clearly indicated the respective area. As a result, 17 studies were categorized as MUAs [28,29,31,32,35,41,44,57,59,61] and 31 as non-MUAs [25,26,27,30,33,34,42,43,45,46,47,48,49,50,51,52,53,54,55,56,58,60]. In terms of MUAs, the effect of RBPM on SBP showed a WMD of 3.213 mmHg (1.521–4.905, *p <* 0.001), with I^2^ = 48.904% (*p* = 0.012, Tau^2^ = 2.793), indicating a moderate degree of between-study heterogeneity based on Cohen’s rule of thumb. In contrast, in non-MUAs, the effect of RBPM on SBP showed a WMD of 5.224 mmHg (3.878–6.569; *p <* 0.001), with I^2^ = 73.152% (*p <* 0.001, Tau^2^ = 12.943). 

#### 3.4.3. Duration of Intervention

**Figure A3:**
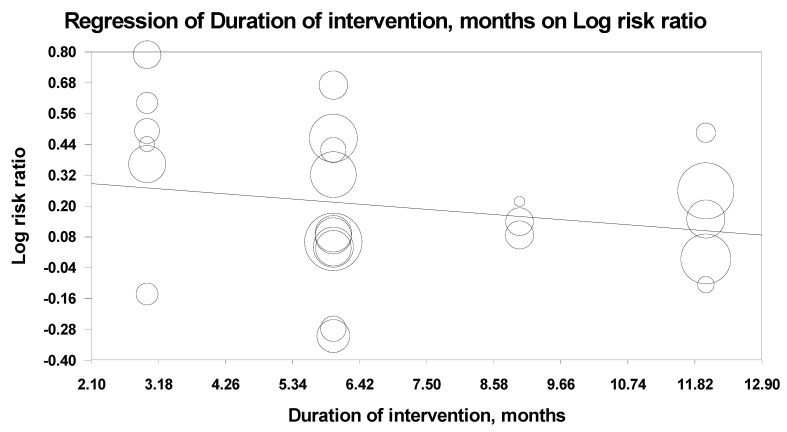
Note: point estimate of individual study (○); RBPM, remote blood pressure monitoring.

The effect of reduced SBP based on the WMD varied according to the duration of the intervention. For an intervention duration ≤3 months [27,28,29,30,33,34,49,51,52,54,58,60], the effect was a WMD of 6.219 mmHg (*n* = 15, 3.970–8.468, *p <* 0.001; I^2^ = 70.060, *p <* 0.001). For 6 months [26,27,28,29,30,32,33,35,42,45,47,48,55,59], the effect was a WMD of 4.491 mmHg (*n* = 14, 2.461–6.521, *p <* 0.001; I^2^ = 84.562, *p <* 0.001). For 12 months, the effect was a WMD of 3.446 mmHg (*n* = 12, 1.209–5.683, *p =* 0.003; I^2^ = 34.656, *p* = 0.113). The rate of BP control had an RR of 1.540 (*n* = 6, 1.223–1.939, *p <* 0.001) after 3 months [27,30,33,51,52,54], an RR of 1.159 (*n* = 11, 1.002–1.341, *p =* 0.047) after 6 months [25,27,30,32,33,45,47,48,59], and an RR of 1.167 (*n* = 5, 0.930–1.464, *p =* 0.183) after 12 months [32,46,50,53,61] (Appendix D, Figure A3).

#### 3.4.4. Setting

The BP reducing effect was analyzed according to the size of the medical institution where RBPM was mainly performed. In primary care clinics, the WMD was 2.981 mmHg (*n* = 14, 1.323–4.639, *p <* 0.001; I^2^ = 45.343, *p =* 0.034) [25,35,44,45,47,48,50,51,53,54,56]. In community health centers, the WMD was 3.512 mmHg (*n* = 12, 1.651–5.373, *p <* 0.001; I^2^ = 31.670, *p <* 0.001) [27,28,29,30,42,57,61], and the WMD at hospital level was 6.333 mmHg (*n* = 22, 4.750–7.917, *p <* 0.001; I^2^ = 73.401, *p <* 0.001) [26,31,32,33,34,41,43,46,49,52,55,58,59,60]. 

#### 3.4.5. Frequency of Remote Transmission of Blood Pressure Data 

In the primary studies in which the frequency of remote BP transmission was reported, when BP information was transmitted daily, the WMD was 5.881 mmHg (*n* = 13, 3.898–7.864, *p <* 0.001; I^2^ = 14.635, *p <* 0.001) [27,34,49,53,54,55,59,60,61]. For weekly BP transmission, the WMD was 4.024 (*n* = 15, 2.641–5.406, *p <* 0.001; I^2^ = 54.610, *p <* 0.001) [28,30,32,42,43,45,47,52,56,57,58]. For biweekly BP transmission, the WMD was 3.941 mmHg (*n* = 4, 1.428–6.454, *p <* 0.001; I^2^ = 0.000). For monthly BP transmission, the WMD was 1.803 mmHg (*n* = 6, −0.234–3.841, *p =* 0.083; I^2^ = 21.639, *p =* 0.056) [26,35,41,50].

## 4. Discussion

The development of healthcare infrastructure and physicians’ preference for practice in an urban setting implies higher accessibility to healthcare and higher patient satisfaction regarding healthcare [65]. However, the COVID-19 pandemic has raised concerns regarding face-to-face care in cities being a potential infection route between healthcare professionals and patients. In this study, data published since September 2018 were included and integrated with data from previous studies to undertake an updated analysis. 

Compared with UC, RBPM for urban-dwelling patients with hypertension was found to significantly reduce SBP and DBP in both statistical and clinical terms, while improving the rate of BP control. Following RBPM, SBP and DBP WMDs decreased by 4.464 mmHg and 2.075 mmHg, respectively, compared with UC. This change, observed through quantitative data, showed a greater margin of decrease than reported in a previous meta-analysis (SBP, 3.482 mmHg; DBP, 1.638 mmHg) [20]. Moreover, according to the temporal interval, the decrease in SBP (1.515 vs. 4.719 mmHg) and DBP (2.059 vs. 2.438 mmHg) in phase III was significantly greater than that in phase I. Therefore, we consider that the demand for RBPM has increased in line with technological advancements, the increased use of mobile devices, and the acceptance of new technologies [66].

RBPM is frequently used in pilot projects preceding the full launch of telemedicine, as it is relatively simple and cost-effective compared with other types of telemedicine. However, reports on the effect of RBPM on the rate of BP control have been inconsistent across numerous previous studies [14]. In this study, where additional data were comprehensively analyzed to extend the meta-analysis, RBPM led to an approximately 20% higher rate of BP control than UC. This is a greater magnitude of improvement than the 13% figure reported in a previous analysis [20]. Considering that the rate of BP control is <50% in traditional face-to-face care, even in countries with advanced healthcare systems, an improvement of 20% is indicative of a highly significant contribution to the prevention of CVD [67]. 

The ultimate objective behind attempts to lower and control BP in patients with hypertension and to bring it closer to a target BP is to reduce the incidence of CVD. However, in the meta-analysis in this study, data were not analyzed in relation to cardiovascular (CV) events because the included RCTs primarily showed outcomes that targeted changes in BP or the rate of BP control, not CV events. Nevertheless, the effect of RBPM on CV events in urban-dwelling patients with hypertension can be conjectured based on the results of previous studies. In a previous large-scale meta-analysis on prospective monitoring, including randomized, controlled, placebo trials or anti-hypertensive studies, a decrease of 2–3 mmHg in SBP in patients with a moderate risk of CVD was shown to cause a 10% reduction in CV mortality and a 20–30% reduction in major adverse CV events [2,68,69,70]. Thus, the observed decrease in SBP of 4.464 mmHg in this study, when the WMD was compared between UC and RBPM, is clinically significant and potentially contributes to reducing CV events.

The effect size of the primary outcomes was set as the SMD and, as it showed moderate-to-high heterogeneity (I^2^ = 70.908%; *p <* 0.001), a subgroup analysis was performed (Appendix E, Table A1). First, the analysis according to city size (based on population size) showed that the effect of RBPM was greater in cities with a population of ≥1 million (SBP, 3.860 mmHg, *p <* 0.001; I^2^ = 0.000, *p* = 0.478) than in small-to-medium cities with a smaller population, although within-study heterogeneity was high (I^2^ = 82.177, *p <* 0.001). The effect of RBPM in reducing SBP was statistically significant compared with UC, irrespective of city size. The rate of BP control also showed greater effects in large cities (RR, 1.268; *p <* 0.001) than in small-to-medium-sized cities (RR, 1.157; *p =* 0.094). In a previous literature review, the intervention effect was found to be smaller in larger cities (large city, 3.229 mmHg vs. small-to-medium city, 3.765 mmHg), where the difference was considered to be associated with the difference in technological utility based on acceptance [66]. In particular, there was a sudden rise in demand for telemedicine to avoid the transmission of infectious diseases in response to the COVID-19 pandemic in 2020 [71,72]. 

Second, subgroup analysis was also performed according to urban MUAs in terms of healthcare accessibility. The decrease in BP in relation to RBPM in non-MUAs was 5.224 mmHg (I^2^ = 73.152%, *p <* 0.001), indicating a greater effect of RBPM in reducing SBP compared with MUAs (3.213 mmHg, *p <* 0.001; I^2^ = 48.904%, *p* = 0.012). The extent to which the level of within-study heterogeneity affects the summary effect size remains unclear, but the results of the analysis provided supporting evidence for determining the overall effect. Although a precise reason for this result could not be identified in this study, the following factors may be considered: changes in attitudes towards the use of mobile devices and chronic disease management and changes in economic lifestyle related to reduced opportunities for healthcare. These results may be used as evidence by healthcare policy-makers to support the need for differentiated policies for the supply of telemedicine in urban settings.

Third, a subgroup analysis was also performed concerning the duration of intervention. No optimal schedule has been established for the period of management of hypertension based on RBPM and the frequency of remote transmission of data [47,73]. Despite slight differences in the magnitude of reduction in SBP, RBPM in this study showed a consistent effect of reducing SBP, regardless of duration. Nonetheless, as the intervention duration increased, the level of BP reduction decreased. The reason for such a decrease could not be clearly identified, but possible causes may be fatigue, indifference, and inadequate level of perceived utility due to the prolonged performance of the intervention [73,74]. However, considering that it is essential to achieve a target BP as early as possible in patients with hypertension to prevent CVD, the effect of RBPM on the early outcome of BP reduction may be emphasized for its use in practice. The optimal duration of RBPM should be limited to a short period of time due to hypertension being a chronic disease requiring long-term management.

Fourth, in this updated study, subgroup analysis was undertaken according to the setting where RBPM was mainly implemented. Accordingly, when the intervention was performed at a tertiary hospital, RBPM had a significant reduction in BP (6.33 mmHg, *p <* 0.001; I^2^ = 73.401%, *p <* 0.001). The same numerical comparison was not compared in each group and, in the case of hospitals, its size was not analyzed separately; however, the results were statistically significant and included a sufficient number of studies to support the results; therefore, the significance of the results should not be ignored. The reason that RBPM had a higher BP lowering effect in tertiary medical institutions than in primary medical institutions may be due to the greater financial and human resource capacity in tertiary medical institutions [75]. 

Finally, this study observed the effect of RBPM with respect to the frequency of transmission of BP data. In the case of daily transmission, the WMD decreased by 5.881 mmHg. In contrast, in the case of monthly transmission, a decrease of 1.803 mmHg was observed. Some conflicting studies show that the higher the frequency of remote transmission, the lower the BP reduction effect [60,61]. However, in our study on cities, the longer the transmission interval, the lower the effect. 

In previous meta-analyses, the number of studies conducted in urban settings was insufficient, and no study showed a change according to temporal progression. In this updated research, we included a comparison of the average effect over time, which was not covered in previous studies, and the effect according to the frequency of setting and data teletransmission. In particular, in our previous meta-analysis, it was reported that the effect of RBPM on patients with hypertension in metropolitan cities was not as large as that in small and medium cities. However, in this updated study, we found that the decrease in SBP and DBP was large in cities with a population of ≥1 million. Therefore, this study addressed the limitations of previous studies. Advancements in telecommunication technology have led to increased use of remote monitoring in healthcare [76]. In situations where physical distancing is emphasized, such as in the case of COVID-19, it is essential to assess the effects of RBPM in an urban setting [77]. To our knowledge, this study is the first meta-analysis to assess the effects of RBPM in urban-dwelling patients with hypertension from RBPM inception to the end of November 2020, including during the COVID-19 pandemic period, and these comprehensive results may provide a clinical basis for developing future healthcare policies.

In this study, a structured formula was applied, and a transparent process was followed to analyze RCTs with a high level of evidence. However, this study had some limitations. First, although the final studies were selected through a structured search using reliable databases, there may have been a language barrier. No outlier was found to have an influence on the summary effect size through the “one-study removed” sensitivity test method and a cumulative meta-analysis; however, selecting articles in different languages may have prevented adequate accounting for errors. Although most abstracts included in the search were written in English, the collected data may not have been sufficient. To overcome this limitation, multiple languages need to be set in the search with a wider scope to include gray literature. Second, the number of small-sized articles was insufficient to test for publication errors. Egger’s test for the results in this study was used to determine combined two-tailed *p*-value significance, and the number of articles with a nil result in terms of a 95% CI was as high as 2898, which increased reliability. Nevertheless, there remained the possibility of publication errors. This limitation could be addressed through including a larger number of small-sized articles. Third, as the studies included in this meta-analysis varied in terms of the period when they were conducted, the criteria for target BP reflected in the rate of BP control may also have varied. Thus, further studies should set a clear BP target for collecting and synthesizing the data to produce more accurate results. Fourth, the authors categorized time intervals to compare the average SBP according to time interval and to quantify the results, which involved dividing the studies according to time based on the year 2000, when internet use expanded globally, and making simple comparisons at 10-year intervals thereafter. However, distinctions between time intervals may have been unclear. Although it is not possible to clearly divide the development time of telemedicine technology, we consider that the timeframe could be set more precisely based on historical developments in mobile communication technology and telemedicine. Finally, we examined trends in the effect of RBPM over time through categorizing studies based on their publication dates to indicate the temporal association with COVID-19. However, since differences between the actual dates of research and publication dates are possible, a future study should clarify the dates during which studies were conducted or include more studies published after the onset of the COVID-19 pandemic to address this limitation.

## 5. Conclusions

Our study findings indicated that RBPM for urban-dwelling patients with hypertension was a practical and clinically effective means of reducing office BP. As the cumulative analysis shows, a consistent and clear effect was found in terms of reduction in office SBP following RBPM according to the temporal progress of the primary studies included in this study; an identical trend was found for 2020. 

Based on the primary findings, the effects were classified according to intervention duration, city size, setting, frequency of remote monitoring of BP data, and urban MUAs, and it is anticipated that the implementation of specific policies in relation to these factors would more effectively guide the application of efficient and successful urban remote monitoring. Future studies should analyze more specific variables and include a greater number of studies to obtain more reliable results.

## Figures and Tables

**Table 1:** Characteristics of individual primary studies.

Study	Included Participants	Participants Number		Participants’ Age Interval (Years)			Duration (Months)	City Name (Country)	Population of City	Setting	Description of Intervention	Intervention Frequency	Outcomes
		UC	RBPM	Age Interval	UC	RBPM							
Bosworth (2007) [25]	Treated hypertensive patients	150	150	Child, Adult, Older Adult	Not reported	Not reported	18	Durham (USA)	232,299 in 2005	Durham VA general internal medicine clinics (Not underserved)	Nurse-administered tailored behavioral intervention with telemedicine device connected to telephone	Once a day	1. Primary: BP control. 2. Secondary: knowledge and perceived risks related with hypertension
Bosworth (2007) [25]	Treated hypertensive patients	150	150	Child, Adult, Older Adult	Not reported	Not reported	18	Durham (USA)	232,299 in 2005	Durham VA general internal medicine clinics (Not underserved)	Nurse-administered medication management	Once a day	1. Primary: BP control 2. Secondary: knowledge and perceived risks related with hypertension
Bosworth (2007) [25]	Treated hypertensive patients	150	150	Child, Adult, Older Adult	Not reported	Not reported	18	Durham (USA)	232,299 in 2005	Durham VA general internal medicine clinics (Not underserved)	Nurse-administered tailored behavioral intervention and medication management	Once a day	1. Primary: BP control 2. Secondary: knowledge and perceived risks related with hypertension
Kerry (2013) [26]	Hypertensive patients with history of stroke or transient ischemic attack	169	168	16 or older (Child, Adult, Older Adult) Average: 71.9	72.6 ± 11.4	71.1 ± 12.6	6	London (UK)	6,984,772 in 2007	Community healthcare center (Not underserved)	Home BP monitoring with nurse-led support through telephone	Twice a week	Reduction of SBP
Kerry (2013) [26]	Hypertensive patients with history of stroke or transient ischemic attack	169	168	16 or older (Child, Adult, Older Adult) Average: 71.9	72.6 ± 11.4	71.1 ± 12.6	12	London (UK)	6,984,772 in 2007	Community healthcare center (Not underserved)	Home BP monitoring with nurse-led support through telephone	Twice a week	Reduction of SBP
Pan (2018) [27]	Patients diagnosed hypertension	55	52	Between 35 and 75. Average: 57.2	56.55 ± 9.80	57.8 ± 10.87	3	Beijing (China)	11,895,973 in 2016	Fangzhuang Community Health Center (Not underserved)	Mobile phone-linked computer system	Once a day	BP control
Pan (2018) [27]	Patients diagnosed hypertension	55	52	Between 35 and 75. Average: 57.2	56.55 ± 9.80	57.8 ± 10.87	6	Beijing (China)	11,895,973 in 2016	Fangzhuang Community Health Center (Not underserved)	Mobile phone-linked computer system	Once a day	BP control
Zha (2020) [28]	Uncontrolled hypertensive patients	13	12	Between 18 and 64. Average: 52.3	55.5 ± 5.2	48.9 ± 8.0	3	Newark (USA)	278,366 in 2016	Jordan and Harris Community Health Center (Local community health center) (Underserved)	Smartphone-linked system by nurse	Visit office once a week. Instant feedback after all measurements.	BP control (Changes in SBP and DBP), perceived self-efficacy, HRQOL
Zha (2020) [28]	Uncontrolled hypertensive patients	13	12	Between 18 and 64. Average: 52.3	55.5 ± 5.2	48.9 ± 8.0	6	Newark (USA)	278,366 in 2016	Jordan and Harris Community Health Center (Local community health center) (Underserved)	Smartphone-linked system by nurse	Visit office once a week. Instant feedback after all measurements.	BP control (Changes in SBP and DBP), perceived self-efficacy, HRQOL
Artinian (2007) [29]	African American hypertensive patients	157	164	18 or more	60.2 ± 12.3	59.1 ± 13.0	3	Detroit (USA)	594,562 in 2002	Family community center (Underserved)	Telephonic transmission with BP monitoring device linked to telephone	Once a week	Office BP changes (SBP, DBP)
Artinian (2007) [29]	African American hypertensive patients	163	168	18 or more	60.2 ± 12.3	59.1 ± 13.0	6	Detroit (USA)	594,562 in 2002	Family community center (Undeserved)	Telephonic transmission with BP monitoring device linked to telephone	Once a month	Office BP changes (SBP, DBP)
Artinian (2007) [29]	African American hypertensive patients	169	167	18 or more	60.2 ± 12.3	59.1 ± 13.0	12	Detroit (USA)	594,562 in 2002	Family community center (Undeserved)	Telephonic transmission with BP monitoring device linked to telephone	Once a month	Office BP changes (SBP, DBP)
Cicolini (2013) [30]	Treated or untreated hypertensive patients	98	100	Between 18 and 80. (Adult, Older Adult) Average: 59.1	58.3 ± 13.9	59.8 ± 15.0	3	Chieti (Italy)	43,824 in 2011	Italian Hypertension Primary Care Center (Not underserved)	Nurse-led reminder through e-mail	Once a week	1. BP changes 2. BMI, alcohol consumption, cigarette smoking, adherence to therapy
Cicolini (2013) [30]	Treated or untreated hypertensive patients	98	100	Between 18 and 80. (Adult, Older Adult) Average: 59.1	58.3 ± 13.9	59.8 ± 15.0	6	Chieti (Italy)	43,824 in 2011	Italian Hypertension Primary Care Center (Not underserved)	Nurse-led reminder through e-mail	Once a week	1. BP changes 2. BMI, alcohol consumption, cigarette smoking, adherence to therapy
Hebert (2012) [31]	Uncontrolled hypertensive patients	83	85	18 or more. Average: 60.8	(61.3 ± 11.7)	61.3 ± 11.7	9	New York (USA)	8,174,959 in 2010	One academic medical center, two medium-sized hospitals, one community hospital (Underserved)	Telephone	Once a week (Meetings: once in two weeks)	Blood pressure reduction
Hebert (2012) [31]	Uncontrolled hypertensive patients	78	79	18 or more. Average: 60.8 Average: 60.8	(61.3 ± 11.7)	61.3 ± 11.7	18	New York (USA)	7,721,457 in 2010	One academic medical center, two medium-sized hospitals, one community hospital (Underserved)	Telephone	Once a week (Meetings: once in two weeks)	Blood pressure reduction
Kim (2014) [32]	Uncontrolled Korean–American hypertensive seniors	192	191	60 or older adult. Average: 70.9	71.2 ± 5.6	70.6 ± 5.0	6	Ellicott City (USA)	60,489 in 2007	Korean Resource Center (Hospital) (Not Undeserved)	Telephone-monitoring system and telephone counseling	At least once a week (Measurement: at least twice a day, Monthly telephone counseling)	Changes in SBP and DBP
Kim (2014) [32]	Uncontrolled Korean–American hypertensive seniors	185	187	60 or older adult. Average: 70.9	71.2 ± 5.6	70.6 ± 5.0	12	Ellicott City (USA)	60,489 in 2007	Korean Resource Center (Hospital) (Not Undeserved)	Telephone-monitoring system and telephone counseling	At least once a week (Measurement: at least twice a day, Monthly telephone counseling,	Changes in SBP and DBP
Kim (2014) [32]	Uncontrolled Korean–American hypertensive seniors	185	184	60 or older adult. Average: 70.9	71.2 ± 5.6	70.6 ± 5.0	18	Ellicott City (USA)	60,489 in 2007	Korean Resource Center (Hospital) (Not Undeserved)	Telephone-monitoring system and telephone counseling	At least once a week (Measurement: at least twice a day, Monthly telephone counseling,	Changes in SBP and DBP
Mohsen (2020) [33]	Treated hypertensive patients with antihypertensive medication	50	50	Between 35 and 65. Average: 56.41	55.01 ± 7.50	57.81 ± 9.52	3	Shibin El Kom (Egypt)	190.064 in 2019	Medical outpatient clinic of Menoufia University Hospital (Not Undeserved)	Tele-nursing intervention with telephone support	Twice a week (Measurement: every day)	1. Reduction of SBP and DBP 2. BMI difference
Mohsen (2020) [33]	Treated hypertensive patients with medication	50	50	Between 35 and 65. Average: 56.41	55.01 ± 7.50	57.81 ± 9.52	6	Shibin El Kom (Egypt)	190.064 in 2019	Medical outpatient clinic of Menoufia University Hospital (Not Undeserved)	Tele-nursing intervention with telephone support	Twice a week. (Measurement: every day)	1. Reduction of SBP and DBP 2. BMI difference
Pour (2020) [34]	Treated hypertensive patients with medication	21	21	Between 35 and 64. Average: 55.7	56.71 ± 5.73	54.71 ± 6.11	3	Tehran (Iran)	7,250,693 in 2019	Military hospital (Not underserved)	Interactive SMS	Once a week	BP control (Changes in SBP and DBP),
Pour (2020) [34]	Treated hypertensive patients with medication	21	21	Between 35 and 64. Average: 55.7	56.71 ± 5.73	54.71 ± 6.11	4	Tehran (Iran)	7,250,693 in 2019	Military hospital (Not underserved)	Interactive SMS	Once a week	BP control (Changes in SBP and DBP),
Pour (2020) [34]	Treated hypertensive patients with medication	21	21	Between 35 and 64. Average: 55.7	56.71 ± 5.73	54.71 ± 6.11	3	Tehran (Iran)	7,250,693 in 2019	Military hospital (Not underserved)	Non-Interactive SMS	Once a week	BP control (Changes in SBP and DBP),
Pour (2020) [34]	Treated hypertensive patients with medication	21	21	Between 35 and 64. Average: 55.7	56.71 ± 5.73	54.71 ± 6.11	4	Tehran (Iran)	7,250,693 in 2019	Military hospital (Not underserved)	Non-Interactive SMS	Once a week	BP control (Changes in SBP and DBP),
Rubinstein (2016) [35]	Untreated prehypertensive patients	276	270	Between 30 and 60. Average: 43.4	43.2 ± 8.4	43.6 ± 8.4	6	Buenos Aires (Argentina) and Guatemala City (Guatemala) and Lima (Peru)	12,271,254 (Buenos Aires) and 880,893 (Guatemala City) and 7,136,586 (Lima) in 2012	Institute for Clinical Effectiveness and Health Policy (Buenos Aires, Argentina), Institute of Nutrition of Central America and Panama (Guatemala City, Guatemala), Universidad Peruana Cayetano Heredia (Lima, Peru) (Underserved)	Mobile phone transmission	Once a month	Mean changes in SBP and DBP
Rubinstein (2016) [35]	Untreated prehypertensive patients	287	266	Between 30 and 60. Average: 43.4	43.2 ± 8.4	43.6 ± 8.4	12	Buenos Aires (Argentina) and Guatemala City (Guatemala) and Lima (Peru)	12,271,254 (Buenos Aires) and 880,893 (Guatemala city) and 7,136,586 (Lima) in 2012	Institute for Clinical Effectiveness and Health Policy (Buenos Aires, Argentina), Insitute of Nutrition of Central America and Panama (Guatemala City, Guatemala), Universidad Peruana Cayetano Heredia (Lima, Peru) (Underserved)	Mobile phone transmission	Once a month	Mean changes in SBP and DBP
Hill (1999) [41]	Black or African American hypertensive young male residents within hospital catchment area	77	78	Between 22 and 49 Average: 39.0			12	Baltimore (USA)	503,998 in 1995	Johns Hopkins Hospital Outpatient General Clinical Research Center (Underserved)	Telephone	Once a month	Office BP changes
Friedman (1996) [42]	Treated hypertensive patients	134	133	Over 60 Average: 76.5	77	76	6	Boston (USA)	534,743 in 1994	Senior centers in 29 different communities(Not underserved)	Telephone-linked computer system	Once a week	Office BP changes
McMahon (2005) [43]	Poorly controlled diabetics and hypertensive patients	35	37	Older than 18. Average: 63.5	63 ± 7	64 ± 7	12	Boston (USA)	580,352 in 2001	Hospital (Not underserved)	Web-base	At least three times a week	Changes in A_1c_, BP, lipid profiles
Shea (2006) [44]	Diabetic hypertensive patients	347	333	55 or older (Adult, Older Adult) Average: 70.8 ± 6.7	70.9 ± 6.8	70.8 ± 6.5	12	Syracuse (USA)	129,966 in 2005	SUNY Upstate Medical University hospital, (Underserved)	Telephone-linked web system	Regularly	Changes in hemoglobin A_1c_, BP, cholesterol level
Carrasco (2008) [45]	Treated or untreated hypertensive patients	142	131	Average age: 62.5	62.8 ± 12.5	62.1 ± 11.9	3	Madrid (Spain)	3,116,909 in 2006	21 regional public health centers (the corporative network of the “Servicio Madrileno de Salud”) (Not underserved)	Mobile phone transmission	During the six-month follow-up, four times a week (Monday and Thursday, morning and night)	1. BP control 2. the impact on patient QoL and anxiety, and economic aspects concerning the viability of the telemedicine system
Green (2008) [46]	Treated hypertensive patients	247	246	Between 25 and 75. (Adult, Older Adult) Average: 59.1	58.6 ± 8.5	59.5 ± 8.3	12	Seattle, USA	622,927 in 2006	10 medical centers within Group Health Research Institute (Not underserved)	Home BP monitors, instruction on their use, and proficiency training on web-based communication	Report once every two weeks (measurement at least twice a week)	Office SBP and DBP changes and control of BP
Madsen (2008) [47]	Treated or untreated hypertensive patients	123	113	Between 20 and 80. Average Age: 55.9	56.7 ± 11.6	55.0 ± 11.7	6	Holstebro (Denmark)	29,888 in 2004	Holstebro Hospital (Not underserved)	PDA-embedded mobile-web phone (mobile)	Three times a week during the first 3 months and once a week during the last 3 months	Difference in systolic daytime ABPM change
Parati (2009) [48]	Uncontrolled hypertensive patients	111	187	Between 17 and 75. Average age: 57.5	58.1 ± 10.8	57.2 ± 10.7	6	Milan (Italy)	1,198,182 in 2006	Primary care units in Milan (Not underserved)	Telephone-linked computer system	Regularly	Percentage of patients who reached normalization of BP
Park (2009) [49]	Obese hypertensive patients	21	28	Average age: 53.8	54.6 ± 11.0	53.2 ± 6.9	2	Seoul (S. Korea)	9,828,102 in 2007	University-affiliated tertiary care hospital (Not underserved)	Telephone and internet transmission	Once a week	Change in blood pressure, body weight, waist circumference, and serum lipid profile
Varis (2010) [50]	Untreated hypertensive patients	68	89	Between 40 and 80	Not reported	Not reported	13	Helsinki and Tampere and Turku (Finland)	536,160 and 194,594 and 168,920 In 2007	Not underserved	Letter to physician	Every five weeks (measurement every day)	Changes in BP and target BP
Hoffmann-Petersen (2017) [51]	Treated uncomplicated hypertensive patients	181	175	Between 55 and 64 Average: 60.4	60.4 ± 2.9	60.5 ± 2.6	3	Holstebro (Denmark)	30,885 in 2011	Holstebro Regional Hospital (Not underserved)	Telephone and e-mail communication (Telephone-linked computer system)	Once every two weeks	Daytime ABPM reduction and percentage of target BP
Ionov (2020) [52]	Uncontrolled hypertension patients	80	160	Between 18 and 78	49 (20 to 77)	47 (18 to 78)	3	Saint-Petersburg, (Russia)	5,076,520 in 2019	Federal Medical Research Center Hospital (Not underserved)	Mobile phone communication	Once a week (Measurement: twice a day)	Change of SBP and rate of BP control.
Logan (2012) [53]	Uncontrolled hypertensive and diabetic patients	51	54	30 or more Average: 62.9	62.7 ± 7.8	63.1 ± 9.0	12	Toronto (Canada)	2,423,221 in 2011	Mount Sinai Hospital (Not underserved)	Bluetooth-enabled BP device paired with smartphone (mobile-web)	Twice a day	Changes in ambulatory BP
Neumann (2011) [54]	Inadequately treated hypertensive patients	29	28	Between 18 and 80. Average age: 55.5	56.2 ± 17.4	54.7 ± 17.9	3	Göttingen (Germany)	119,161 in 2009	Not underserved	Mobile phone-linked computer system	Once a Day	BP Control
Wakefield (2011) [55]	Type 2 diabetics and hypertensive patients	97	83	Between 40 and 89. Average: 48.1	67.9 ± 9.9	68.4 ± 9.5	6	Iowa City (USA)	67,548 in 2006	Iowa City VA Health Care System (Not underserved)	Telephonic transmission	Every day	Changes in hemoglobin A_1c_ and SBP
Bosworth (2011) [56]	Treated hypertensive patients	137	127	Child, Adult, Older Adult Average Age: 63.5	64 ± 10	63 ± 11	12	Durham (USA)	234,477 in 2006	Durham VA Medical Center (Not underserved)	Telephonic transmission	Once a day	1. BP control 2. SBP and DBP change
Migneault (2012) [57]	African American hypertensive patients	140	125	35 or more. Average age: 56.5	56.8 ± 11.4	56.3 ± 10.6	8	Boston (USA)	590,971, in 2003	Boston Medical Center primary care practices of a large, safety-net hospital and four affiliated community health centers. (Underserved)	Automated, computer-based, interactive telephone counseling system	Once a week	Change in diet quality, leisure time physical activity of moderate-or-greater intensity, and adherence to the antihypertensive medication regimen and change in BP.
Park (2012) [58]	Post-menopausal obese hypertensive patients	33	34	Average age: 56.7	57.6 ± 5.5	55.8 ± 5.7	3	Seoul (S. Korea)	9,828,102 in 2007	University medical center (Not underserved)	Reporting on website. Mobile and internet transmission	Once a week.	Change in waist circumference, body weight, and blood pressure, fasting plasma glucose, and serum lipid levels
Bove (2013) [59]	Systolic hypertensive patients	107	99	Between 18 and 85 (Adult, Older Adult) Average: 59.6	58.2 ± 13.5	61.0 ± 13.6	6	Philadelphia/Wilmington, USA	1,480,457/109,499 in 2010	University hospital (Underserved)	Telephone and internet-based System	Once a day	BP control at 6 months
Wakefield (2014) [60]	Type 2 diabetics and uncontrolled hypertensive patients	43	40	18 or more. Average: 60.0	62.5 ± 10.9	57.7 ± 10.8	3	Columbia (USA)	112,498 in 2010	University hospital (Not underserved)	Web System through mobile phone or personal computer	Twice a week (Measurement: every day)	Changes in hemoglobin A_1c_ and SBP
Yi (2015) [61]	Uncontrolled hypertensive patients	332	329	18 or more. Average: 61.3	61.3 ± 12.2	61.3 ± 11.9	9	Bronx and Brooklyn and New York (USA)	1,308,242 and 2,172,989 and 7,721,458 in 2010	Riverdale Family Practice (Bronx), Lutheran Family Health Centers (Brooklyn), New York City Department of Health and Mental Hygiene (New York City), Heritage Health Care (New York City) (Underserved)	Telephone-linked computer system	Once a month (Measurement: every day)	Change in SBP and DBP and achievement of BP control

Abbreviations: ABPM, ambulatory blood pressure monitoring; A_1__c_, glycated hemoglobin; BMI, body mass index; BP, blood pressure; DBP, diastolic blood pressure; HRQOL, health-related quality of life; QoL, quality of life; RBPM, remote blood pressure monitoring; SBP, systolic blood pressure; SMS, short message service; UC, usual care.

## Data Availability

Not applicable.

## Supplementary Materials

The following are available online at www.mdpi.com/article/10.3390/ijerph182010583/s1, Table S1: PRISMA 2020 Checklist.

## Appendix A. Searching Strategy via Cochrane Library

1. MeSH descriptor: [Hypertension] explode all trees
2. hypertensi* OR high blood pressure
3. OR/1,2
4. MeSH descriptor: [Urban population] explode all trees
5. MeSH descriptor: [Urban health] explode all trees urban health [Mesh]
6. MeSH descriptor: [Urban health services] explode all trees
7. MeSH descriptor: [Cities] explode all trees
8. urban* OR city OR cities OR central cit*
9. OR/4–8
10. AND/3,10
11. MeSH descriptor: [Telemedicine] explode all trees
12. MeSH descriptor: [Telemetry] explode all trees
13. MeSH descriptor: [Blood pressure monitoring, ambulatory] explode all trees
14. telemedicine OR telemetry OR telenurs* OR telemonitor* OR eHealth OR telehealth OR remote monitor* OR technolog* OR telephone OR smartphone OR internet
15. OR/12–15
16. AND/11,16
17. randomised controlled trial OR randomized controlled
18. controlled clinical trial
19. randomised [tiab] OR randomized [tiab]
20. 2placebo [tiab]
21. drug therapy [sh]
22. groups [tiab]
23. clinical trials as topic [tiab]
24. randomly [tiab]
25. trial [tiab]
26. OR/18–26
27. 27 NOT cluster randomized controlled trials
28. 28 NOT cross over study
29. AND/17,29

## Appendix E. Subgroup Analysis

**Table A1:** CI, confidence interval; FEM, fixed effects model; SBP, systolic blood pressure; WMD, weighted mean difference.

Category	Number of Studies	Summary WMD of SBP, mmHg (95% CI)	Heterogeneity, I^2^ (%) Using an FEM (*p*-Value)	Heterogeneity, Tau-Squared (τ^2^) Using an FEM
Overall	48	4.464 (3.371–5.556)	70.908 (*p* < 0.001)	9.200
City size (population)				
<1 million	22	3.860 (2.271–5.450)	0.000 (*p* = 0.478)	0.000
>1 million	26	5.056 (3.503–6.609)	82.177 (*p* < 0.001)	17.368
Medically underserved areas				
Underserved	17	3.213 (1.521–4.905)	48.904 (*p* = 0.012)	2.793
Not underserved	31	5.224 (3.878–6.569)	73.152 (*p* < 0.001)	12.943
Duration (month)				
≤3	15	6.198 (4.019–8.377)	70.060 (*p* < 0.001)	14.069
6	14	4.479 (2.524–6.433)	84.562 (*p* < 0.001)	17.240
9	4	2.116 (-1.816–6.048)	0.000 (*p* = 0.752)	0.000
12	12	3.436 (1.281–5.591)	34.656 (*p* = 0.113)	1.646
Setting				
Primary care clinic	14	2.981 (1.323–4.639)	45.243 (*p* = 0.034)	1.989
Community health center	12	3.512 (1.651–5.373)	31.670 (*p* = 0.138)	1.883
Hospital	22	6.333 (4.750–7.917)	73.401 (*p* < 0.001)	17.133
Frequency of data transmission				
Daily	13	5.881 (3.898–7.864)	14.635 (*p* = 0.297)	1.637
Weekly	15	4.024 (2.641–5.406)	53.610 (*p* = 0.007)	4.505
Bi-weekly	4	3.941 (1.428–6.454)	0.000 (*p* = 0.622)	0.000
Monthly	6	1.803 (-0.234–3.841)	21.639 (*p* = 0.271)	0.552
